# Ultra-brief Assessment of Working Memory Capacity: Ambulatory Assessment Study Using Smartphones

**DOI:** 10.2196/40188

**Published:** 2023-01-27

**Authors:** Jonathan G Hakun, Nelson A Roque, Courtney R Gerver, Eric S Cerino

**Affiliations:** 1 Department of Neurology The Pennsylvania State University College of Medicine Hershey, PA United States; 2 Department of Psychology The Pennsylvania State University University Park, PA United States; 3 Center for Healthy Aging The Pennsylvania State University University Park, PA United States; 4 Translational Brain Research Center The Pennsylvania State University College of Medicine Hershey, PA United States; 5 Department of Psychology University of Central Florida Orlando, FL United States; 6 Department of Psychological Sciences Northern Arizona University Flagstaff, AZ United States

**Keywords:** ecological momentary assessment, EMA, mobile cognitive assessment, working memory capacity, measurement burst design, mobile phone

## Abstract

**Background:**

The development of mobile technology with substantial computing power (ie, smartphones) has enabled the adaptation of performance-based cognitive assessments to remote administration and novel intensive longitudinal study designs (eg, measurement burst designs). Although an “ambulatory” cognitive assessment paradigm may provide new research opportunities, the adaptation of conventional measures to a mobile format conducive to intensive repeated measurement involves balancing measurement precision, administration time, and procedural consistency.

**Objective:**

Across 3 studies, we adapted “complex span” tests of working memory capacity (WMC) for ultra-brief, smartphone-based administration and examined their reliability, sufficiency, and associations with full-length, laboratory-based computerized administrations.

**Methods:**

In a laboratory-based setting, study 1 examined associations between ultra-brief smartphone adaptations of the operation span, symmetry span, and rotation span tasks and full-length computerized versions. In study 2, we conducted a 4-day ecological momentary assessment (EMA) study (4 assessments per day), where we examined the reliability of ultra-brief, ambulatory administrations of each task. In study 3, we conducted a 7-day EMA study (5 assessments per day) involving the ultra-brief rotation span task, where we examined reliability in the absence of extensive onboarding and training.

**Results:**

Measurement models in study 1 suggest that comparable estimates of latent WMC can be recovered from ultra-brief complex span task performance on smartphones. Significant correlations between the ultra-brief tasks and respective full-length versions were observed in study 1 and 2, ranging from *r*=0.4 to *r*=0.57. Results of study 2 and study 3 suggest that reliable between-person estimates of operation span, symmetry span, rotation span, and latent WMC can be obtained in 2-3 ultra-brief administrations (equivalent to <1 day of testing in an EMA study design). The results of study 3 replicated our findings, showing that reliable between-person estimates of rotation span may be obtained in as few as 2 ultra-brief administrations in the absence of extensive onboarding and training. In addition, the modification of task parameterization for study 3 improved the estimates of reliability of within-person change.

**Conclusions:**

Ultra-brief administration of complex span tasks on smartphones in a measurement burst design can generate highly reliable cross-sectional estimates of WMC. Considerations for future mobile cognitive assessment designs and parameterizations are discussed.

## Introduction—General

The proliferation of mobile technology with substantial processing power has enabled a significant leap forward for intensive longitudinal study designs aiming to examine psychological and behavioral processes under naturalistic circumstances. This active area of research is often referred to as ecological momentary assessment (EMA) and leverages pervasive mobile technology (eg, tablets and smartphones) to study how these processes, which have most often been well studied under controlled laboratory conditions, unfold over the course of daily life [1,2]. Recently, the translation of computerized cognitive assessments to administration on mobile devices has increased the potential for integrating performance-based measures of cognition into EMA study designs [3,4]. This emerging paradigm, often referred to as “mobile” or “ambulatory” cognitive assessment, offers an opportunity to deploy studies of cognition at scale (through remote, phone- or tablet-based administration) and to study the role of cognitive variation in processes that unfold over multiple timescales. Existing web- and mobile app–based platforms have confirmed the feasibility and validity of remote delivery in various populations [5-7]. However, a major hurdle in adapting performance-based assessments of cognition to intensive study designs involves the need to conform task and assessment parameters to unsupervised, ultra-brief administrations. In this paper, we present the results of 3 studies that examined the feasibility of integrating “complex span” task assessments of working memory capacity (WMC) into intensive longitudinal study designs on mobile phones.

WMC describes an individual’s ability to maintain information in short-term memory while processing ongoing or new information [8]. WMC is often approached as a domain-general cognitive construct indicated by performance on “complex” short-term memory span tasks covering multiple stimulus domains (eg, verbal, visual, spatial, etc; [9]). Individual differences in WMC are strongly associated with reasoning ability or fluid intelligence (Gf) [10,11], indicators of attention and executive control [8,12-14], and the ability to retrieve information displaced from the focus of attention [15].

Recent applied research also suggests that WMC may play an important role in goal-directed behavioral processes. For example, in laboratory-based experiments, individuals with higher WMC have been shown to exhibit more successful self-regulation (eg, behave more consistently with stated behavioral standards; [16,17]). In addition, studies linking laboratory-based assessment of WMC with EMA of stress-related processes indicate that individual differences in WMC may predict emotion regulation ability, as indicated by negative affective responses to stressor exposures over the course of daily life [18]. Critically, these examples of work connecting WMC to complex behavioral processes are based on single laboratory-based assessments of WMC, which have limited examination of the role of WMC in these processes to the level of interindividual differences. Recent evidence suggests that WM function may vary substantially within individuals within and between days [19-21], and the development of ambulatory assessments of WMC would enable investigations into the role of intraindividual variation in WMC in processes that unfold over time (eg, behavior change processes [22]).

Intensive longitudinal study designs commonly used in EMA research, such as the “measurement burst design,” involve sampling multiple times per day over multiple days to generate a rich description of contexts, behavior, or experiences under study [23,24]. This design offers several advantages over single or more periodic assessments. Sampling in close temporal proximity to an event or behavior may help reduce retrospective bias and other forms of confounding common to self-report methods (eg, schematic responding), develop a more accurate description of experience through aggregation of momentary data, and allow researchers to unravel time-dependent associations between the factors under study [25].

The measurement advantages associated with EMA may also extend to ambulatory performance-based cognitive assessments. Aggregation of data over many ambulatory cognitive assessments can mitigate the influence of a single episode of poor or inspired performance, which may otherwise misrepresent an individual’s overall average ability (ie, reduce the impact of temporal sampling error; [26]). The increased ecological validity associated with completing assessments in the context of a person’s natural environment (eg, at home) may reduce the negative effects of novel testing conditions (eg, clinics and laboratories). High-frequency cognitive assessments may provide valuable insights into cognitive development and disease by revealing the degree to which cognition systematically varies over moments, days, and contexts [27]. Finally, ambulatory cognitive assessment promises the ability to liberate assessment procedures from the laboratory and clinic context and reach participant populations geographically separated from these resources and assessment opportunities (ie, improve the generalizability of research by bringing data collection to participants). However, achieving the goal of implementing performance-based assessments in EMA research designs requires the ability to obtain reliable estimates of cognition in just a few minutes through ultra-brief adaptations of cognitive testing paradigms. In the current set of studies, we sought to evaluate whether ultra-brief adaptations of complex span tasks could provide reliable span estimates of WMC under different study design parameters and constraints.

The paper is organized into 3 studies. In the first study, we describe the results of a laboratory-based study designed to examine the degree to which scores on ultra-brief adaptations of the operation span (OSpan), symmetry span (SSpan), and rotation span (RSpan) tasks, administered on smartphones, are correlated with full-length, computerized versions and determine whether latent estimates of WMC can be recovered from performance on ultra-brief tasks. In the second study, we describe the results of a measurement burst design study in which we assessed the reliability of the 3 ultra-brief tasks and composite WMC across intensive repeated administrations occurring over 4 days. In the third study, we describe the results of an EMA field study involving the ultra-brief Rotation Span task to illustrate task psychometrics in a context in which participants received minimal in-laboratory practice and training with the task procedures.

## Study 1

### Introduction

In study 1, we conducted a cross-sectional study of performance on automated full-length OSpan, SSpan, and RSpan tasks and delayed performance on smartphone-based ultra-brief adaptations of each task (an average of 3 days later in a separate session). We examined correlations between ultra-brief and full-length task performance for each complex span task, the fit of confirmatory measurement models, and criterion correlation with a Gf factor indicated by performance on 2 reasoning tasks.

### Methods

#### Participants

A total of 132 younger adults (female: 95/132, 72%; mean age 19.12, SD 1.05, range 18-23 years) participated in this study. Participants were recruited from the psychology undergraduate research pool at the Pennsylvania State University.

#### Ethics Approval

All the participants provided written informed consent and received course credit for their participation. All experimental procedures were approved by Pennsylvania State University’s institutional review board for the ethical treatment of human participants (reference number STUDY00003499).

#### Materials and Procedure

##### Experimental Measures

Participants completed 3 full-length, automated complex span tasks (OSpan, SSpan, and RSpan) and 2 reasoning tasks (Ravens Advanced Progressive Matrices, RAPM and Letter Sets Task, Letters) in a 90-minute laboratory session. Tasks were administered in a fixed order across all participants to control for potential crosstalk between tasks: OSpan, SSpan, RSpan, RAPM, and Letters. Following the initial laboratory session, participants were scheduled to return to the laboratory for a brief session, an average of 3 days later (range 2-4), where ultra-brief versions of the OSpan, SSpan, and RSpan tasks (“amb-OSpan,” “amb-SSpan,” and “amb-RSpan,” respectively) were administered on a smartphone provided by the laboratory (Samsung Galaxy S5; Samsung Electronics).

##### Full-Length Complex Span Tasks

###### OSpan Task

OSpan involves memorizing a single letter at a time while performing interleaved arithmetic operations. After memorizing a set of letters and completing the interleaved arithmetic operations, the participants were asked to recall all of the letters memorized throughout the current trial in order. Trials randomly varied in set-size. Set-sizes (# of total letters tested in each trial) ranged from 3 to 7, with 3 repetitions of each set-size throughout the task. The total number of item operation pairs was 75.

###### SSpan Task

SSpan involved memorizing the highlighted locations in a 4×4 matrix one at a time while performing interleaved symmetry judgments on 8×8 mosaic pattern stimuli that were either symmetrical or nonsymmetrical along the vertical axis. After memorizing a set of locations and completing the interleaved symmetry judgments, participants were asked to recall all spatial locations from the 4×4 matrices memorized throughout the current trial in order. Trials randomly varied in set-size. Set-sizes (# of total locations tested in each trial) ranged from 2 to 5, with 3 repetitions of each set-size throughout the task. The total number of location symmetry pairs was 42.

###### RSpan Task

RSpan involved memorizing oriented arrows one at a time while performing interleaved mental rotation judgments on letter stimuli that were either forward or backward facing if oriented back to standard typeface orientation. Study arrows were oriented at 45° increments around an invisible clock face and were either short or long in total length, allowing for 16 possible orientation-length combinations. After memorizing a set of arrows and completing the interleaved mental rotation judgments, participants were asked to recall all of the oriented arrows memorized throughout the current trial in order. Trials randomly varied in the set-size. Set-sizes (# of total arrows tested in each trial) ranged from 2 to 5, with 3 repetitions of each set-size throughout the task. The total number of arrow rotation pairs was 42.

###### Automated Task Procedures and Scoring

Each automated task contained self-guided instructions, practice, and timing parameters customized to the participant (Redick et al [28] and Conway et al [29] provide a full description of the development, parameters, and reliability of these automated procedures). Each automated task was scored according to the partial-trial scoring method. The partial-trial scoring method involves counting each item recalled from memory regardless of whether all items within a trial were recalled. The partial-trial scoring method was selected to equate scoring methods between the full-length and ultra-brief versions of the tasks, where only 3 trials were administered in the latter.

##### Reasoning Tasks

###### RAPM Task

RAPM evaluated 3×3 matrices of patterned tiles, with a tile missing in the lower right-hand corner of each matrix. For every matrix, participants were asked to select a sample tile from 8 possible choices located at the bottom of the screen that they believed would best complete the overall pattern present in the matrix (the patterns included horizontal and vertical progressions). Participants were given 10 minutes to complete as many matrices as possible before the assessment timed out. The number of correct responses obtained over the course of 10 minutes served as the RAPM score.

###### Letters

Letters evaluated 5 groups of 4 letters each with a pattern or rule governing the association among 4 of the 5 groups. For every set of letters, participants were asked to select the group that violated the pattern or rule implied by the other 4 groups. Participants were given 10 minutes to complete as many sets as possible before the assessment timed out. The number of correct responses obtained over the course of 10 minutes served as the Letters score.

##### Ultra-brief Complex Span Tasks

**Figure 1:**
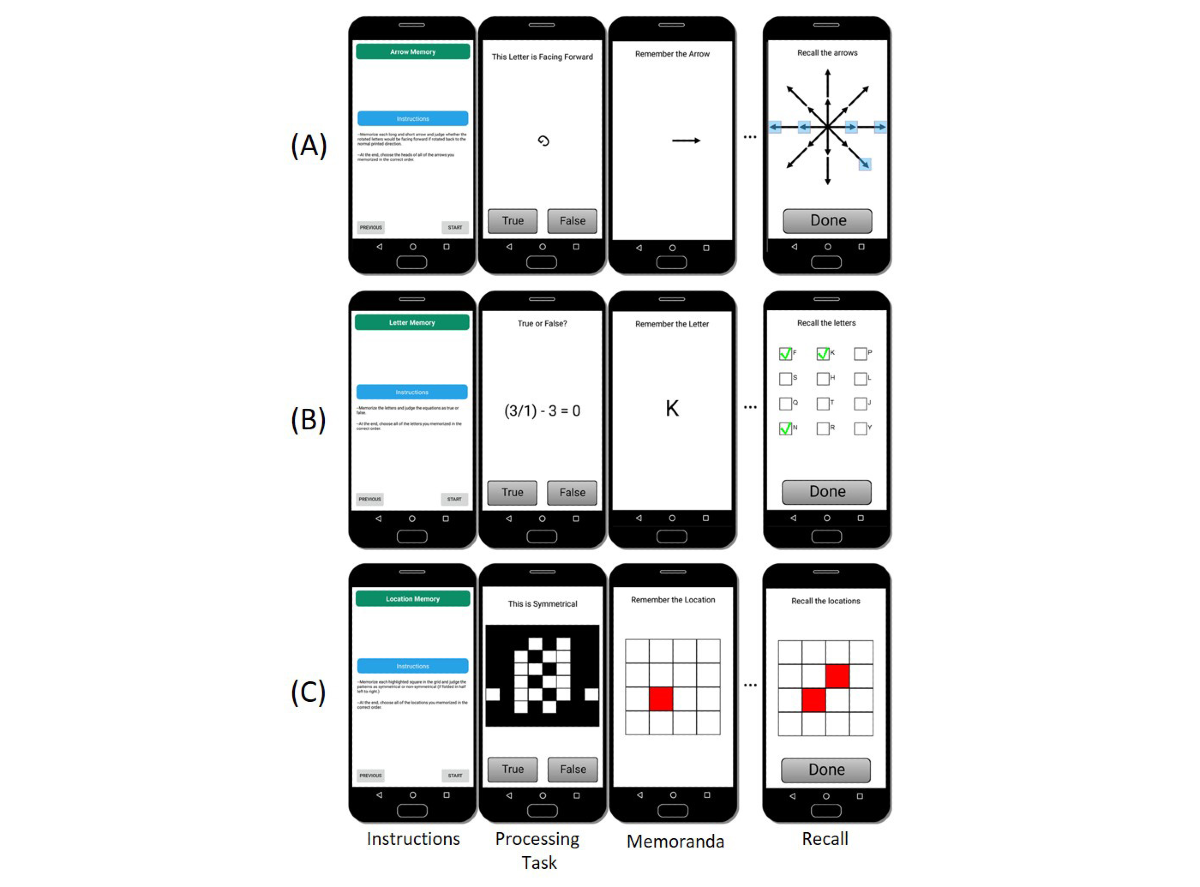
Smartphone-adapted complex span tasks. (A) Ultra-brief rotation span task (“amb-RSpan”). (B) Ultra-brief operation span task (“amb-OSpan”). (C) Ultra-brief symmetry span task (“amb-SSpan”). Images presented in the figure are screen captures of each task as presented via the Mobile Monitoring of Cognitive Change (“M2C2”) mobile app used in studies 2 and 3.

Ultra-brief, smartphone-adapted versions of the full-length, automated complex span tasks used in study 1 were programmed using Qualtrics survey software (Qualtrics) and administered using the Qualtrics “Offline Surveys” smartphone app (Qualtrics) installed on the investigator-provided Samsung Galaxy S5 phones. The ultra-brief tasks were presented in a fixed order across participants, consistent with the order of the full-length task presentation in the first session: amb-OSpan, amb-SSpan, and amb-RSpan. The participants completed 3 trials for each task, one at each set-size. Set-sizes for the amb-OSpan task ranged from 4 to 6, and set-sizes for the amb-SSpan and amb-RSpan tasks ranged from 3 to 5. The scale of set-sizes selected for the ultra-brief tasks (ie, higher set-sizes for amb-OSpan than for the other 2 tasks) was set to reflect the scale of the full-length tasks. The ultra-brief tasks contained no practice phase and included only an instruction screen that reminded the participants of the respective task procedures. Owing to limitations in the degree to which set-size randomization could be achieved using Qualtrics, within each task, set-sizes were presented in a pseudorandomized and counterbalanced order across all participants. Participants logged their responses using a touch screen (Figure 1). The total administration time for all 3 tasks was approximately 4.5 minutes, or approximately 30 seconds per trial.

#### Statistical Analyses

Confirmatory factor analyses and structural equation modeling analyses were performed using the Mplus software package (version 8.1; Muthén & Muthén [30]; using maximum likelihood estimation). The factor goodness of fit was evaluated using chi-square fit statistics, root mean square error of approximation (RMSEA), standardized root mean squared residual (SRMR), and comparative fit indices (CFI [31,32]). Nonsignificant chi-square values, RMSEA values ≤0.08, SRMR ≤0.08, and CFI >0.9 generally serve as indications of acceptable fit [31,32]. All fit indices were considered when determining goodness of fit.

### Results

#### Descriptive Statistics

Means, SDs, and first-order correlations for each task are shown in Table 1. Significant correlations were observed between each of the full-length complex span tasks and the respective ultra-brief phone-based tasks (eg, OSpan and amb-OSpan; *r=*0.40-0.57, *Ps*<.05). Generally, ultra-brief tasks were significantly correlated with nonmatching domain full-length tasks (eg, SSpan and amb-RSpan). However, a marginal correlation was observed between OSpan and amb-SSpan (*r=*0.15, *P*=.09).

**Table 1:** Correlations between full-length and ultra-brief tasks in study 1.

Variable	1	2	3	4	5	6	7	8
OSpan^a^	61.1 (8.7)							
SSpan^b^	0.36	29.6 (7.8)						
RSpan^c^	0.32	0.50	27.9 (8.2)					
amb-OSpan	0.40	0.21	0.18	14.3 (1.1)				
amb-SSpan	0.15^d^	0.57	0.31	0.19	9.7 (2)			
amb-RSpan	0.31	0.44	0.50	0.24	0.34	9.3 (2)		
RAPM^e^	0.28	0.45	0.44	0.19	0.30	0.33	15.3 (4.5)	
Letters	0.25	0.30	0.34	0.27	0.20	0.47	0.43	15.9 (4.1)

^a^OSpan: operation span.

^b^SSpan: symmetry span.

^c^RSpan: rotation span.

^d^All correlations in the table above are significant, *P*<.05, except correlation between OSpan and amb-SSpan was marginal, *P*=.09. Values on the diagonal are mean (SD).

^e^RAPM: Ravens Advanced Progressive Matrices.

#### Confirmatory Measurement Models

**Figure 2:**
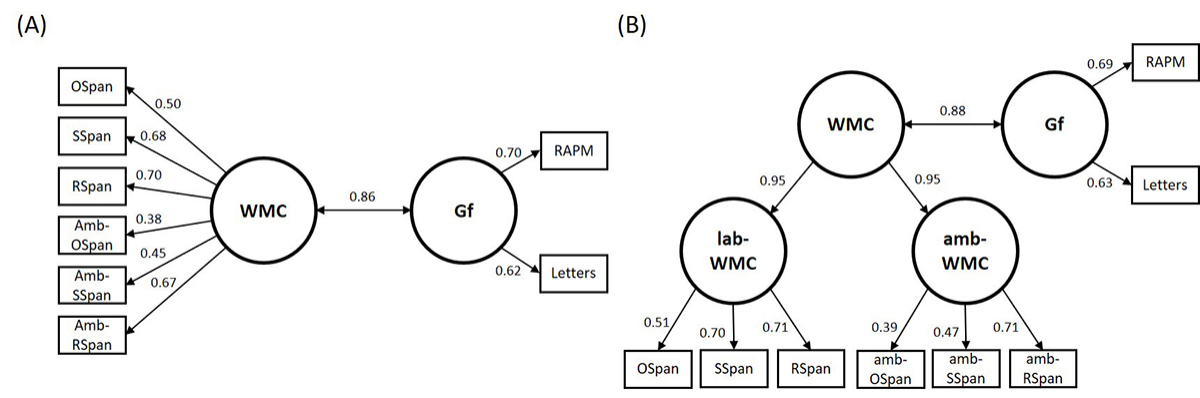
Measurement models including full-length and ultra-brief tasks. (A) Model 1, one-factor model of WMC indicated by full-length in-laboratory and ultra-brief phone-based complex span tasks. (B) Model 2, hierarchical factor model of WMC indicated by 2 subordinate latent WMC factors representing full-length in-laboratory complex span tasks (lab-WMC) and ultra-brief phone-based complex span tasks (amb-WMC). WMC: Working Memory Capacity; Gf: fluid intelligence.

We considered 2 measurement models. Model 1 included a one-factor representation of WMC, where the ultra-brief and full-length tasks were all considered indicators of the same latent WMC factor. Model 1 was fit to directly compare factor loadings between administration modalities (full-length and ultra-brief) on a common latent WMC factor. Model 2 included a hierarchical factor representation where 2 latent factors (lab-WMC and amb-WMC) were indicated by full-length in-laboratory tasks and ultra-brief phone-based tasks, respectively. In model 2, lab-WMC and amb-WMC were modeled as latent indicators of a superordinate WMC factor. Model 2 was fit to determine the relative contribution of each modality-specific WMC factor (lab- and amb-WMC) to the criterion correlation with a latent reasoning factor. In each model, a latent reasoning (Gf) factor was included, as indicated by RAPM and Letters, and correlations between Gf and the highest-level WMC factors were estimated. Both models exhibited good fit to the data (Model 1: χ*^2^*_18_=25.5, *P*=.01; RMSEA=0.056, 90% CI 0.000-0.103; SRMR=0.049; CFI=0.967 and Model 2: χ*^2^*_16_=24.2, *P*=.08; RMSEA=0.062, 90% CI 0.000-0.110; SRMR=0.049; CFI=0.964). The factor loading for the amb-RSpan task in Model 1 was consistent with that for the full-length task. Loadings for amb-OSpan and amb-SSpan were lower than those for the respective full-length tasks. Consistent with previous research, WMC was highly associated with Gf in both models (Figure 2). The results of model 2 indicated that the association between WMC and Gf observed in model 1 was not biased by the contribution of the full-length task indicators (equal subordinate WMC factor loadings were observed).

#### Sensitivity Analysis

Participants were most accurate on the lowest set-size trials for each ultra-brief task (set-size 4 for amb-OSpan; set-size 3 for amb-SSpan and amb-RSpan). Participants accurately responded to 93% of all set-size 4 trials for the amb-OSpan task, 74% of all set-size 3 trials for the amb-SSpan task, and 70% of all set-size 3 trials for the amb-RSpan task. Performance on higher set-size trials yielded less skewed distributions. The inclusion of low set-size trials was consistent with standard procedures for the automated full-length tasks. Given our goal of developing assessments that are as brief as possible, following the method used by Oswald et al [33], we refit model 1 by removing the lowest set-size trial from each ultra-brief task. The revised version of model 1 exhibited good fit to the data (χ*^2^*_18_=29.7, *P*=.04; RMSEA=0.07, 90% CI 0.015-0.114; SRMR=0.058; CFI=0.940). Factor loadings and correlations between WMC and Gf in the revised model were highly consistent with model 1 (all observed loadings and correlations were within 0.05 of model 1 parameters).

### Discussion

The results of study 1 suggest that estimates of complex span task performance obtained via ultra-brief assessments on smartphones correlate with full-length, computerized administrations. The results of the confirmatory measurement models suggest that latent estimates of WMC can be recovered from performance on ultra-brief complex span tasks (although lower factor loadings were observed for the amb-OSpan and amb-SSpan tasks than for the full-length versions). Consistent with previous studies, a strong association between latent WMC and Gf was observed. The results of the sensitivity analysis suggest that estimates of latent WMC may be obtained in as few as 3 minutes of administration on smartphones (2 trials of each task, 30 seconds per trial). Building on this observation, in study 2, we examined the psychometric properties of 2-trial, ultra-brief complex span tasks in the context of a measurement burst study design.

## Study 2

### Introduction

A primary motivation for the development and psychometric assessment of ultra-brief complex span tasks on smartphones is to implement these tasks in EMA study designs (eg, measurement bursts). Measurement burst designs typically involve intensive repeated assessments; for example, 3-5 assessments per day, over a period of a few days to weeks. Measurement bursts can be scheduled in a manner that allows for a comparable, or greater, amount of testing time or trials to be accumulated over repeated ultra-brief administrations as would be conducted during a single lab-based session. In support of cross-sectional study designs, in study 2, we examined the number of ultra-brief assessments necessary to generate reliable cross-sectional estimates of WMC (ie, between-person reliability) in the context of an unsupervised measurement burst. In this case, we refer to between-person reliability as reflecting the consistency of mean performance across a fixed *N* administration of ultra-brief tasks (see *Statistical Analysis* section). Incremental estimates of between-person reliability would be useful to prospectively determine the number of administrations necessary to power projects and analyses focused on *individual differences* in WMC. Estimates of between-person reliability are often calculated based on data collected from the same individual at multiple occasions. However, between-person reliability is distinct and statistically independent of estimates of within-person reliability [34].

As mentioned previously, another advantage of adapting performance-based assessments of cognition to EMA is the ability to capture and model short-term variation in cognition within individuals over time, with the goal of examining within-person processes that might depend upon, or interact with, WMC. Here, we refer to within-person reliability in terms of the ability of ultra-brief tasks to generate reliable estimates of short-term changes in WMC that occur across measurement occasions (eg, between moments or days). To estimate between- and within-person reliability of ambulatory, ultra-brief complex span task performance, in study 2, we followed the procedures described by Cranford et al [35] to conduct generalizability and decision studies based on Generalizability Theory [36].

### Methods

#### Participants

A total of 39 younger adults (female: 28/39, 72%; mean age 20.64, *SD* 3.01; range 18-30 years) participated in the study. Participants were recruited from the psychology undergraduate research pool at the Pennsylvania State University and through local advertisements.

#### Ethics Approval

All the participants provided written informed consent and received course credit or compensation for their participation. All experimental procedures were approved by Pennsylvania State University’s institutional review board for the ethical treatment of human participants (reference number STUDY00003499).

#### Materials and Procedure

##### Experimental Procedures

Participants were acquainted with the task procedures for each complex span task by completing abbreviated versions of the automated complex span tasks used in study 1 (OSpan, SSpan, RSpan) using parameters described by Foster et al [37] and Oswald et al [33] during a 60-minute laboratory session. Tasks were administered in a fixed order across all participants to control for potential cross-talk between tasks: OSpan, SSpan, RSpan, and followed by full-length administrations of the RAPM and LETTERS tasks, as described in study 1. Following the laboratory session, participants were asked to carry a laboratory-provided smartphone for 4 days. A custom prototype Android mobile app currently under development for the National Institutes of Health (NIH) Mobile Toolbox (the “Mobile Monitoring of Cognitive Change” or “M2C2” app) was used to administer the battery of ultra-brief complex span tasks and generate 4 notifications per day requesting participants to begin the assessments. The first notification of each day was scheduled to occur within 2 hours of the participant’s self-reported average waking time. Subsequent notifications were separated by an average of 3.75 hours and pseudorandomly jittered around this interval by up to 30 minutes.

##### M2C2 Adaptation of Ultra-brief Complex Span Tasks

Ultra-brief (“amb-“) versions of the OSpan, SSpan, and RSpan tasks were programmed on the M2C2 platform and administered on Samsung Galaxy S5 smartphones (Samsung Electronics). The ultra-brief tasks were presented in a fixed order across participants, consistent with the order of the full-length task presentation in the laboratory session: amb-OSpan, amb-SSpan, and amb-RSpan. The participants completed 2 trials of each task, one at each set-size. Consistent with study 1, set-sizes for the amb-OSpan task were 5 and 6 and set-sizes for the amb-SSpan and amb-RSpan tasks were 4 and 5. The ultra-brief tasks contained no practice phase and included only an instruction screen that reminded the participants of the respective task procedures. Within each task, the set-sizes were presented in a random order. The participants logged their responses using the touch screen. The total administration time for all 3 tasks was approximately 3 minutes, or approximately 30 seconds per trial.

#### Statistical Analyses

##### Correlations Between Ambulatory and Laboratory-Based Measures

Participants were highly compliant with the assessment protocol, completing a mean of 15 “*sessions*” (momentary administrations of the ultra-brief task battery) over the 4-day measurement burst. Accordingly, all correlational and psychometric analyses were conducted during the first 15 sessions. Composite WMC scores were generated for each administration modality by *z*-scoring performance on each task and averaging by administration modality (lab-WMC and amb-WMC). Composite Gf scores were generated by *z*-scoring and then averaging performance on the RAPM and LETTERS tasks.

##### Variance Decomposition and Psychometrics

First, as an initial descriptive stage of analysis, 2-level random effects, intercept-only multilevel models were fit to composite amb-WMC and task-specific span scores to describe the proportion of variance between-persons relative to the total variance between- + within-persons (intraclass correlations, ICCs).

Next, to estimate between- and within-person reliability, generalizability studies (“G-study”) were conducted, where burst level (ie, 15 sessions) data were decomposed into systematic and random variance components. The G-studies involved decomposing span score variance across 15 sessions into systematic and random components in a random effects repeated measures ANOVA in R (R Foundation for Statistical Computing; *lme4* package; [38]). Systematic variance associated with persons (*p*), occasions (*o*), items (*i*), and all 2-way interactions (*p×o, p×i, o×i*) were modeled in the repeated-measures ANOVA, along with residual variance (the random component). Separate G-studies of amb-OSpan, amb-SSpan, amb-RSpan, and composite amb-WMC performance were conducted. For the G-studies of amb-Ospan, amb-SSpan, and amb-RSpan, within each session, scores could only vary across the 2 trials presented per task. Thus, the item (*i*) factor for each task-specific G-study was parameterized to account for *trial*-related variance. For the G-studies of composite amb-WMC, trials were first summed within each session by task so that the item (*i*) factor for the G-study of composite amb-WMC was parameterized to account for *task*-related variance. Two G-studies were conducted for composite amb-WMC, enabling the examination of within-person reliability at different timescales, one defining occasion at the session-level (G-momentary) and the other defining occasion at the day-level (G-daily). Decision studies (“D-study”), that is, estimation of reliability at between- and within-person levels, were conducted using formulas described in detail by Cranford et al [35] (Equations 4 and 5; also see Scott et al [39]).

The between-person reliability (*R_BP_*) for each ultra-brief complex span task and composite amb-WMC was calculated in D-studies of incremental epoch lengths of the measurement burst (similar to the correlational analysis described above). *R_BP_* of each ultra-brief task and of composite WMC was calculated based on the first 2 sessions, the first 3 sessions, the first 4 administrations, and so on, up to the 15th session based on the following formula (Equation 4 from Cranford et al [35]):



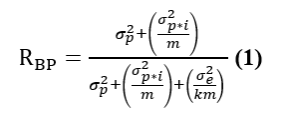



where *σ^2^_p_* is the total systematic variance associated with persons, σ^2^_p*i_ is the total systematic variance associated with the interaction of persons×items (*tasks* for amb-WMC and *trials* for each ultra-brief span task), *σ^2^_e_* is the random residual variance, *m* is the total number of items included in a single occasion (3 *tasks* for the amb-WMC D-Studies and 2 *trials* for the ultra-brief span task D-Studies), and *k* is the total number of sessions included in each D-study.

Within-person reliability of change (*R_WP_*) for composite amb-WMC was calculated at the level of momentary change (*R_WPM_*) and daily change (*R_WPD_*) using the following equation (Equation 5 in Cranford et al [35]):



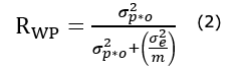



where σ^2^_p*o_ is the total systematic variance associated with the interaction of persons×occasions, *σ^2^_e_* is the random residual variance, and *m* is the total number of items (*task* administrations) within the defined occasion. The results of G-Momentary were used to calculate *R_WPM_*, and the results of G-daily were used to calculate *R_WPD_*. The total number of task administrations within *o* was 3 for *R_WPM_* (one administration of each ultra-brief span task) and 12 for *R_WPD_* (3 tasks per session×4 sessions per day).

### Results

#### Associations Between Ambulatory and Laboratory-Based Measures

**Figure 3:**
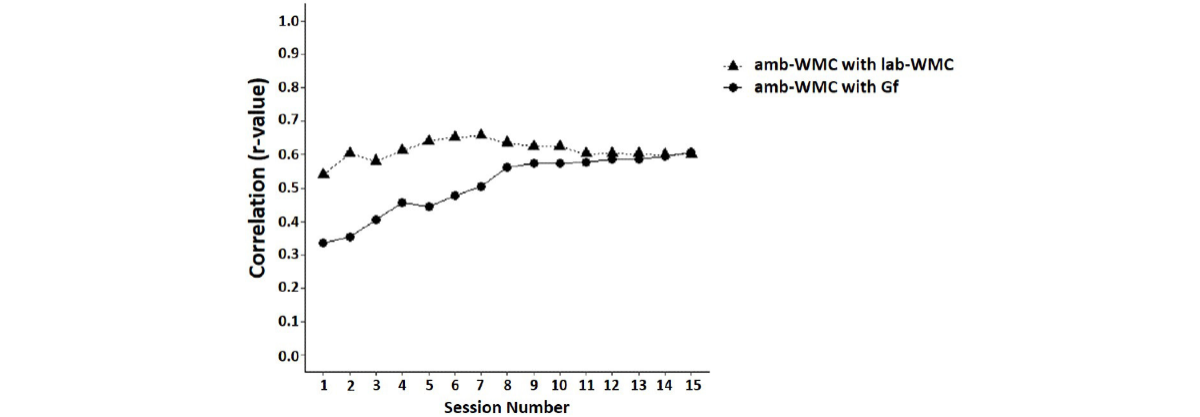
Incremental correlations between amb-WMC, lab-WMC, and Gf. After one session, amb-WMC was significantly correlated with lab-WMC and Gf. After 7 sessions (<2 days of the measurement burst or approximately 21 minutes of total testing) amb-WMC reached a maximum level of correlation with the laboratory-based tasks (r>0.6 with lab-WMC, r>0.5 with Gf). WMC: Working Memory Capacity; Gf: fluid intelligence.

Amb-WMC significantly correlated with both lab-WMC and Gf after the first session (*P*s<.05). Incremental correlation analysis between amb-WMC estimated from sessions 1 to 15 and laboratory-based estimates of composite lab-WMC and Gf suggested that ambulatory and laboratory-based administrations of automated complex span tasks were strongly correlated and reached a maximum level of correlation (*r*=0.66, *P*<.001) after 7 sessions (<2 days of burst administration in the current design). A similar pattern was observed between amb-WMC and Gf, where a correlation of *r>*0.5 (*P*<.001) was observed by the seventh session (Figure 3).

#### ICCs for Composite WMC and Task-Specific Span Scores

Results of a random effects, intercept-only, multilevel model on composite amb-WMC suggest that approximately 47% of the variance observed in amb-WMC over the measurement burst was within persons over time (ICC_amb−WMC_=0.53). Overall, ICCs for task-specific span scores were lower than composite amb-WMC (ICC_amb−OSpan_=0.30; ICC_amb−SSpan_=0.31; ICC_amb−RSpan_=0.39), suggesting greater proportional within-person variance in task-specific span scores than composite amb-WMC.

#### Between-Person Reliability

**Figure 4:**
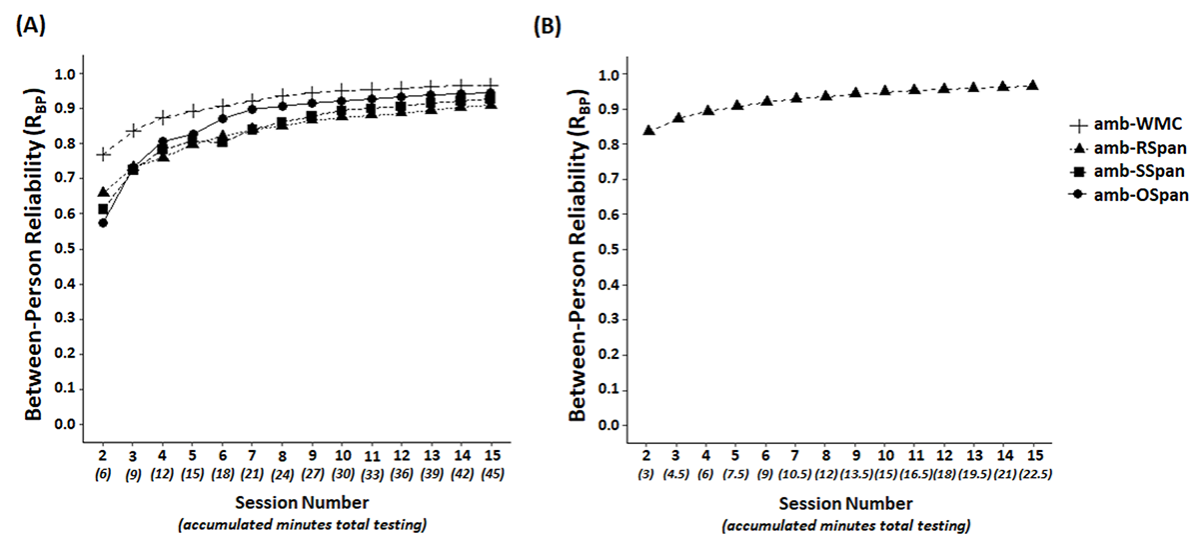
Incremental between-person reliability estimates. (A) Between-person reliability for each ultra-brief complex span task and composite amb-WMC incremented by session in the measurement burst in study 2. (B) Between-person reliability for the ultra-brief rotation span task incremented by session in the measurement burst in study 3. WMC: Working Memory Capacity.

*R_BP_* was calculated incrementally on task-specific span scores and composite amb-WMC (Table 2 provides the amb-WMC G-study results). All individual tasks exhibited *R_BP_*>0.7 after 3 sessions, which is equivalent to approximately 9 minutes of administration during the first day of the measurement burst. Amb-WMC exhibited *R_BP_*=0.76 after 2 sessions, which is equivalent to approximately 6 minutes of testing (Figure 4A).

**Table 2:** Generalizability and decision study results.

Variance source			Study 2		Study 3
			Composite WMC^a^ (variance)		amb-RSpan (variance)
**G-study momentary**					
	Person	0.871		0.488	
	Occasion^b^	0.008		0.007	
	Item^c^	4.639		0.008	
	Person×occasion	0.094		0.177	
	Person×item	0.381		0.005	
	Occasion×item	0.000		0.000	
	Error	1.409		0.914	
	m (# of items)	3		3	
	*R*_WPM_	0.17		0.37	
**G-study daily**					
	Person	0.887		0.486	
	Occasion^b^	0.016		0.009	
	Item^c^	4.642		0.008	
	Person×occasion	0.057		0.064	
	Person×item	0.377		0.001	
	Occasion×item	0.000		0.000	
	Error	1.451		1.032	
	m (# of items)	12		15	
	*R*_WPD_	0.32		0.48	
	*R*_BP_ for burst	0.96		0.97	

^a^WMC: working memory capacity.

^b^For momentary models, Occasion=sessions; for daily models, Occasion=day. RCM/RCD=Reliability of within-person change at momentary/daily level.

^c^Study 2 item=*task;* study 3 item=*trial*.

#### Within-Person Reliability of Change

Within-person reliability of change in amb-WMC was examined at 2 levels: reliability of change across sessions (ie, the “momentary” level; *R_WPM_*) and reliability of change across days (*R_WPD_*). Results of D-studies on reliability of change across sessions and days revealed *R_WPM_*=0.17 at the momentary level and *R_WPD_*=0.32 at the daily level.

### Discussion

The results of study 2 suggest that reliable between-person estimates of composite WMC can be obtained in as few as 2 unsupervised administrations of 3 ultra-brief complex span tasks on smartphones, in a measurement burst design, which is consistent with approximately 6 minutes of total testing (30 seconds per trial × 6 trials × 2 administrations). Estimates of between-person reliability were generally lower for the individual complex span tasks than for the composite WMC, but all ultra-brief tasks exhibited >0.7 reliability after only 3 administrations, approximately 3 minutes of performance on each individual task, or approximately 9 minutes of testing to complete all 3 ultra-brief tasks.

Although WMC is typically studied as a stationary cognitive construct, we observed considerable within-person variation in composite WMC and each ultra-brief task over the measurement burst. In study 2, the proportion of total variance in task-specific scores attributable to within-person variation over time was substantial, ranging from approximately 50% for composite WMC to 70% for the amb-OSpan task. The results of our incremental reliability analysis, however, suggest that the ultra-brief tasks exhibit high between-person reliability with few administrations despite within-person variation in performance over time. Using a variance decomposition approach described by Generalizability Theory, we further investigated what proportion of this within-person variation should be considered systematic (ie, within-person changes) versus random (measurement error).

Intraindividual variation may be attributed to a number of systematic or random factors [34,35,40]. In a second set of analyses in study 2, we decomposed and parameterized systematic and random variance components in the G- and D-studies. We estimated the reliability of within-person change in composite WMC at 2 timescales: momentary and daily. Results of each D-study suggested, based on our current design and administration characteristics (6 trials per session, 4 sessions per day), that composite WMC assessed via 3 ultra-brief complex span tasks exhibited lower within-person than between-person reliability, particularly at the momentary level. In other words, the ability to detect systematic changes in WMC from moment to moment or from day to day was limited in study 2.

However, these results do not suggest that complex span tasks are not suitable for repeat administration or for detecting short-term, within-person changes. Several factors may influence sensitivity to within-person change, including aspects of study design and task parameterization (eg, number of tasks or trials, observations per day, task difficulty, etc). D-study results from study 2 suggest that doubling the number of “items” administered per day (ie, increasing *m* from 12 to 24) would be expected to increase *R_WPD_* to approximately 0.5. Administering approximately 5 minutes of testing at each of 6 sessions per day to achieve higher levels of reliability would remain consistent with the recommendations for EMA design [41], but potentially prohibit additional forms of measurement central to EMA studies (eg, contemporaneous self-report surveys). In contrast, other task parameters (eg, changes in task difficulty) can be modified to improve sensitivity without greatly affecting the administration time. Task parameterization for study 2 was based on tasks that were largely optimized for the study of between-person differences, which may not be optimal for detecting within-person changes (Nezlek [34]). When preparing for study 3, we considered several aspects of study design and task parameterization.

## Study 3

### Introduction

The ability to detect short-term, within-person changes in cognition is highly dependent on the precision of the scores that can be obtained from each administration. Factors that influence precision include the number of trials administered and calibration of task difficulty to the participant’s ability level. In terms of the number of trials administered, in study 2 we prioritized controlling the total administration time while acquiring multiple indicators of WMC, administering only 2 trials of each task. For study 3, we focused on a single indicator of WMC to afford the ability to administer additional trials. We selected the ultra-brief Rotation Span task because it had the strongest factor loading on latent WMC out of the ultra-brief tasks administered in study 1.

The full-length, computerized complex span tasks involve the administration of trials covering a wide range of set-sizes (eg, trials involving the study of between 2-7 items). This is done to allow fine-grain differences between participants of all ability levels to be appreciated (ie, optimized for detection of cross-sectional or individual differences). Keeping this convention, in study 1 and 2, we varied the set-size across trials. Although this approach may optimize the detection of between-person differences, it may reduce sensitivity to variations that occur within an individual over time. In a hypothetical study involving 3 trials per session, for a participant whose ability exceeds the demands associated with lower set-size trials (ie, consistently achieves perfect accuracy on the 2 lower set-size trials), only 1 trial would be sensitive to short-term change or contributing to the estimation of within-person variation. Designing exclusively around high set-size trials would allow for greater sampling in the range of task difficulty where moderate- to high-performing participants may vary the most, while partial-credit scoring procedures would preserve sensitivity to participants with lower ability. For study 3, we administered 3 set-size 5 trials during each EMA session, which was consistent with the highest set-size administered during the in-person, full-length tasks and the ultra-brief tasks administered in study 1 and 2.

In study 3, we evaluated the reliability of ultra-brief rotation span task performance under conditions that would closely approximate a prototypical research use case for future applied research: a study of stress and academic engagement in college-aged students. A final consideration when designing study 3 included participant onboarding methods. Study 1 and 2 were conducted under more controlled participant onboarding and training conditions than we expect researchers will use in applied settings. In study 1 and 2, gold standard, in-laboratory, automated, computerized versions of each complex span task were administered before phone-based administration. These in-laboratory sessions served 2 purposes: to examine correlations between ultra-brief and full-length tasks and to familiarize participants with the task procedures ahead of phone-based administration. However, in practice, a major goal of ambulatory cognitive assessment is to liberate these procedures from the laboratory. In study 3, we conducted a measurement burst study involving ambulatory administration in the absence of extensive task onboarding and training.

### Methods

#### Participants

A total of 102 younger adults (female: 74/102, 73%; mean age 21, SD 3.4, range 18-44 years) participated in the study. Participants were recruited from undergraduate listservs at the Pennsylvania State University.

#### Ethics Approval

All the participants provided written informed consent and received compensation for their participation. All experimental procedures were approved by Pennsylvania State University’s institutional review board for the ethical treatment of human participants (reference number: STUDY00010536).

#### Materials and Procedure

##### Experimental Procedures

Participants were asked to carry a laboratory-provided smartphone for 7 days (Xiaomi Mi A1 model phones configured with Android One OS, version 8.0; Xiaomi Corporation). The prototype M2C2 app was used to administer EMA self-report surveys followed by 3 trials of the amb-RSpan task. M2C2 made a survey available each morning that participants were asked to self-initiate after waking, and generated 4 pseudorandom notifications throughout each day to complete mid-day surveys. The morning and mid-day surveys each concluded with 3 set-size 5 trials of the amb-RSpan task (the highest set-size used in study 1 and 2) and resulted in a total of five 3-trial amb-RSpan sessions per day. The first notification of each day was scheduled to occur within 2 hours of the participant’s self-reported waking time. Subsequent notifications were separated by an average of 3.75 hours and pseudorandomly jittered around this interval by up to 30 minutes. Data related to the self-report surveys were beyond the scope of this study and will be reported elsewhere.

##### Onboarding and Training

Participants were given a visual overview of the amb-RSpan task via screenshots of the task presented on PowerPoint slides (Microsoft Corporation). The slides contained a depiction of a single amb-RSpan trial and verbal instructions on how to respond to the secondary task and the recall phase using the phone touch screen. After reviewing the slides, the participants were asked to complete one amb-RSpan session (3 trials) on a laboratory-provided smartphone in the presence of the study coordinator. Participants were given an opportunity to ask questions after the completion of the practice trials.

##### Ultra-brief RSpan Task

Procedures for the amb-RSpan task were identical to study 2 except that, based on the results of study 2, 3 trials of equal set-size (set-size=5) were administered during each assessment.

#### Statistical Analyses

Between-person reliability and within-person reliability of change were calculated using the same equations as in study 2. Within-person reliability of change was estimated at the momentary and daily levels. Similar to study 2, the item (*i*) factor in the G studies of amb-RSpan was parameterized to reflect *trial*-related variance.

### Results

#### Variance Decomposition of amb-RSpan Performance

Overall, the participants were highly compliant with the assessment protocol, completing *a mean of* 29 sessions over the 7-day measurement burst (out of 35 possible sessions). All reported results reflect the analysis of the first 29 sessions. The results of a random effects, intercept-only ANOVA on amb-RSpan suggest that approximately 49% of the variance observed in amb-RSpan performance over the measurement burst was within persons over time (ICC_amb−RSpan_=0.51).

#### Incremental Between-Person Reliability

*R_BP_* was calculated for session totals ranging from 2 to 29 sessions (the group mean of the number of sessions completed over the measurement burst). Amb-RSpan exhibited *R_BP_*=0.84 after 2 sessions (3 minutes of testing) and *R_BP_*>0.9 after 5 sessions (1 day, 7.5 minutes of testing; Figure 4B).

#### Within-Person Reliability of Change

Within-person reliability of change was examined at 2 levels: reliability of change across momentary sessions (*R_WPM_*) and reliability of change across days (*R_WPD_*; Table 2 summarizes the G-study results). The results of D-studies on within-person reliability of change across sessions and days suggested higher estimates of within-person reliability at the momentary (*R_WPM_*=0.37) and daily levels (*R_WPD_*=0.48) than those observed for composite amb-WMC in study 2.

### Discussion

The results of study 3 demonstrated that reliable between-person, single-indicator estimates of WMC can be obtained on smartphones in the absence of rigorous computerized training on the task procedures. In addition, increasing the number of trials per session, administering only high set-size trials, and administering an additional session per day improved the within-person reliability of change estimates at momentary and daily levels. The influence of the study design parameters on within-person reliability estimates is informative for future research. Ultimately, these findings increase the promise and feasibility of conducting remotely administered research via ambulatory cognitive assessments on smartphones.

## Discussion—General

### Principal Findings

The results of the 3 studies reported here, evaluating the psychometrics of ultra-brief complex span tasks, suggest that valid and reliable domain-general estimates of WMC can be obtained in approximately 6 minutes on smartphones. Estimates derived from approximately 3 minutes of assessment on smartphones replicated the degree of association between WMC and reasoning task/Gf performance observed with full-length in-laboratory administrations (approximately 75 minutes of testing). Reliable cross-sectional estimates of domain-general WMC were observed after two 3-minute sessions in study 2, and reliable single-indicator estimates were observed after two 1.5-minute sessions in study 3. The results from study 3 also suggested that reliable single-indicator estimates of WMC can be obtained in the absence of rigorous training on the task procedures, opening the possibility for valid and reliable estimation of WMC in future research without face-to-face participant onboarding and training. Finally, estimates of within-person reliability observed in study 2 and study 3 highlighted the importance of study and task parameterization in designing assessment protocols that intend to study short-term, within-person changes and variations.

The results of study 2 and 3 suggest that reliable cross-sectional estimates of complex span task performance may be obtained in ambulatory study designs where data are collected on smartphones over the course of everyday life. These findings are encouraging for efforts to engage underrepresented populations in cognitive research (eg, rural participants, participants in areas or communities without a strong connection to an academic institution, etc). Moreover, the ability to leverage mobile tools to study WMC may help sharpen cross-sectional and long-term change estimates in future studies by harnessing the measurement advantages associated with intensive longitudinal study designs [23,24,26,27].

A second major goal of the current set of studies was to design measures that are sensitive to systematic changes in WMC that occur within individuals over short periods (within and between days). The results of multilevel, random intercept models suggested that at least 50%-70% of the total variance in complex span task performance was observed within individuals over repeated administrations (15 sessions over 4 days in study 2; 29 sessions over 7 days in study 3). The challenge for studies of within-person (ie, “intra-individual”) variation is to parse and detect what could be a fairly weak change signal (differences between individuals in variance across occasions; the p*o interaction term in each G-Study) from a noisy background (measurement error; [34,35,40]). Study 2 indicated low within-person reliability of composite WMC. Following this observation, when planning for study 3, we focused on increasing the precision of the estimates obtained during each EMA session through alteration of the task parameters (number of trials administered and task difficulty).

Variance decomposition of amb-RSpan data in study 3 determined that approximately 11% of the total variance was due to how individuals differ in amb-RSpan performance over occasions (ie, the p*o numerator of the within-person reliability coefficient). Although this was an improvement over study 2, a substantial proportion of the total variance was determined to stem from measurement error, which is summed with p*o in the denominator of the within-person reliability coefficient equation (eg, approximately 57% of amb-RSpan total performance variance over 3 trials×29 occasions; Table 2, study 3, G-study Momentary). One approach to maximizing sensitivity to within-person changes would be to simply measure more at each occasion (ie, capitalize on averaging). However, this approach risks a dramatic increase in measurement burden. For example, it would require approximately 12 trials per session (or approximately 6 minutes of testing) to achieve >0.7 within-person reliability this way.

An alternative approach to increasing within-person reliability involves increasing the precision of each individual measurement (ie, each trial). In the context of the G-studies presented here, the trial-to-trial variation within each session remains unaccounted for by systematic components (including the “item” factor, which only captures effects that are stable across sessions, such as order effects). Thus, the ability to sensitively detect within-person changes is proportional to the degree of trial-to-trial variance within sessions. Therefore, tasks for which the lower bound of trial-to-trial differences are substantially high by design (eg, 20% changes in trial scores per item recalled, or X out of 5 items, as in the parameterization of amb-RSpan used in study 3) may be challenging to parameterize in a manner that prioritizes trial-level precision. It is important to note that these barriers to the detection of short-term within-person cognitive change may attenuate within-person associations, but may not threaten the detection of between-person differences or long-term changes [42,43]. Given the increased focus on intraindividual variation in applied research, the future development of ultra-brief ambulatory cognitive assessments should focus on tasks with high trial-level precision. An opportunity to improve sensitivity to systematic within-person changes in WMC may stem from paradigms involving continuous report recall techniques, which were developed from work evaluating competing theories of working memory representation [44].

### Comparison With Previous Work

In study 1 and study 2, we evaluated the mobile assessment of WMC through the lens of a multi-indicator approach to WMC. The multi-indicator, or latent factor, approach treats WMC as a domain-general construct, potentially reflecting the operation of attention and executive cognitive processes [8,9,45, although see 47]. Applied research over the past 30 years has demonstrated that individual differences in WMC are strongly associated with important domains of higher order cognition, development, and behavior, including Gf and reasoning ability [10,11], control of attention or cognitive control [12,48,49], mind wandering [13,50], and everyday skills such as driving [51]. Recent studies have also focused on the possibility that WMC may index an individual’s capacity to carry out goal-directed behavior, modifying how individuals respond in the presence of an environmental or contextual challenge [52,53].

We shifted to a single-indicator approach for study 3 in response to the same forces that are at play in studies involving in-person data collection and full-length task administration, namely participant burden, time, and study resources. Our goal was to deploy a version of the ultra-brief rotation span task parameterized to improve sensitivity to short-term within-person variation. The results of study 3 suggest that these changes do improve the reliability of within-person changes in complex span task performance. We selected the ultra-brief task that the results of study 1 indicated as the strongest indicator of latent WMC. However, the effect of shifting from a multi-indicator, domain-general approach to estimating WMC using a single indicator should not be overlooked. The measurement models from study 1 demonstrated that latent WMC substantially contributes to ultra-brief rotation span task performance. Yet, task- or domain-specific abilities are also present in the manifest scores. The degree to which this affects the interpretation of future applied research using a single-indicator approach needs to be considered in context (ie, does WMC or visuospatial ability, per se, determine one’s everyday mindfulness, learning ability, or otherwise). Future development research will be needed to determine whether the principles applied in study 3 to increase within-person reliability can be applied to other indicators and deployed as a multi-indicator battery to the same effect.

The validation of ultra-brief complex span tasks suitable for repeated administration represents an important step toward examining the role of intraindividual variation in WMC in psychological and behavioral processes that unfold under naturalistic circumstances. Goals for future research in these areas will need to include a description of *when* WMC predicts behavior (eg, is behavior more well-regulated during moments when WMC is higher for that individual?). We assume that although individuals with higher WMC may excel at criterion tasks in the laboratory or under experimental conditions, they may not *always* engage in such successful patterns of behavior. Moreover, it seems unreasonable to assume that individuals with lower WMC would infrequently or *never* exhibit successful patterns of behavior. Moment-to-moment and day-to-day variation in cognitive function may be driven by a wide range of contextual factors, including, but not limited to, stress, fatigue, pain, and negative affect [19,21,54-56]. Future research should leverage intensive repeated designs to reveal *whether*, *how*, and *when* short-term variation in WMC affects behavioral processes and the potential mechanisms through which higher or lower WMC confers an advantage.

The current set of studies leveraged a new mobile platform under development and sponsored by the NIH and National Institute on Aging. The M2C2 platform is part of the NIH Mobile Toolbox project, and is currently undergoing development, validation, norming, and testing across a wide range of use cases and populations. An early prototype of the M2C2 platform was used to conduct study 2 and study 3, which was validated in other studies involving middle-aged and older adult samples [3,4].

### Limitations

The complex span tasks developed for these studies deviated in a few ways from the most widely distributed automated tasks [28]. Choice of scoring approach (full-credit or partial-credit) is always an important consideration. Partial-credit scores have been shown to have preferable psychometric properties to full-credit scores during full-length administrations [28]. In the current 2-3 trial administration context, partial-credit scoring offers an important advantage for measurement precision (in study 3, span range 0-15 in 1 point increments with partial-credit scoring vs 5 point increments with full-credit scoring). We did not include “don’t know” response options on the recall screens for each task. Smartphone screen real estate is at a premium and in the absence of the “don’t know” response option, participants were allowed to submit fewer items than the set-size during recall. We also did not enforce a time limit on the secondary tasks judgments. In the conventional automated tasks, a response time distribution is generated for secondary task judgments during the practice phase and is used to restrict response times during testing. Because we implemented ultra-brief tasks without a practice phase on the smartphones, we did not include an upper boundary for secondary task judgments. In future work, we intend to explore several approaches to support the verification of testing session validity (confirm sufficient attention-on-task) such as setting an upper limit on secondary task response time, brief catch trials, and other confirmatory task manipulations.

This study involved college-aged younger adults; thus, the results and task parameterization may not be generalizable to other populations. Complex span tasks have, generally, been used to study basic cognitive processes, such as the mechanisms underlying executive control, and less frequently (if at all) as a clinical marker. The necessarily complicated instructions and procedures may make them less appropriate for use in clinical studies involving samples with suspected impairments. Validation studies involving middle-aged and older adults are underway to examine whether similar task parameters as those used here might be appropriate for use with older, cognitively unimpaired participants.

Finally, the strength of the correlations observed between the laboratory and ambulatory administrations may raise several questions about how well the ambulatory administrations replicate what is observed in the laboratory. Although there are several potential sources of imprecision in ambulatory testing that may drive down correlations (eg, ultra-brief administrations, potential for distraction in uncontrolled environments, etc), it stands to reason that similar issues apply to the laboratory-based context. The participants are asked to sit in quiet, controlled laboratory environments for more than an hour, focusing their attention on repetitive procedures, managing fatigue, changes in motivation, and internal distractions throughout the testing period. Although the laboratory-based procedures are well established and validated enough to be considered the “gold standard,” the question of which contextual factors are at play and how much they obscure or enhance measures of cognitive ability or performance in each testing context warrants further consideration and future research.

Given the attempt to create an optimal (eg, quiet, isolated) testing context in the laboratory, laboratory-based scores may be assumed to reflect close to the ideal, or maximum ability level. Whether this score reflects the actual capability brought by individuals to face everyday problem solving and decision making remains unknown. A person’s WMC may be immutable, and each measure may more or less accurately reflect one’s underlying ability. However, another view is that laboratory and ambulatory task scores are equally “valid” measures of WMC, simply reflecting WMC under different contextual constraints (ie, WMC as a range, not a level). Our results indicate that there is systematic variation in WMC that occurs over time. Although this is likely due to various contexts over which participants completed the task administrations, it nonetheless points toward a view of cognitive ability that may be situationally constrained by everyday contextual factors. Which aspect of WMC (eg, range, consistency, maximum level, and so on) is a better predictor of real-world outcomes should be the focus of future research.

### Conclusions

The results of these studies suggest that reliable estimates of WMC can be obtained via ultra-brief adaptations of computerized complex span tasks, in a measurement burst design, via smartphones, under naturalistic circumstances. These findings present a new avenue for research on the role of WMC in psychological and behavioral processes that unfold over the course of daily life. Future work should also focus on the optimization of ambulatory cognitive assessments for detecting short-term intraindividual variation in cognition.

## Abbreviations

CFI: comparative fit indices
EMA: ecological momentary assessment
Gf: fluid intelligence
ICC: intraclass correlation
M2C2: Mobile Monitoring of Cognitive Change
NIH: National Institutes of Health
OSpan: operation span
RAPM: Ravens Advanced Progressive Matrices
RMSEA: root mean square error of approximation
RSpan: rotation span
SRMR: standardized root mean squared residual
SSpan: symmetry span
WMC: working memory capacity
